# Surface Morphology of the Interface Junction of CVD Mosaic Single-Crystal Diamond

**DOI:** 10.3390/ma13010091

**Published:** 2019-12-23

**Authors:** Xiwei Wang, Peng Duan, Zhenzhong Cao, Changjiang Liu, Dufu Wang, Yan Peng, Xiangang Xu, Xiaobo Hu

**Affiliations:** 1 State Key Laboratory of Crystal Materials, Shandong University, Jinan 250100, China; xwang21@126.com (X.W.); 13256998266@163.com (P.D.); wangdufu@163.com (D.W.); 2 Jinan Diamond Technology Co. Ltd., Jinan 250101, China; m13608927651@163.com (Z.C.); owlchj@163.com (C.L.)

**Keywords:** CVD mosaic, single crystal diamond, surface morphology

## Abstract

The diamond mosaic grown on the single-crystal diamond substrates by the microwave plasma chemical vapor deposition (MPCVD) method has been studied. The average growth rate was about 16–17 μm/h during 48 hours’ growth. The surface morphologies of the as-grown diamond layer were observed. It was found that the step flow was able to move across the substrates and cover the junction interface. Raman spectroscopic mapping in the central area of the junction revealed the high stress region movement across the junction interface from one substrate to the other for about 200–400 μm. High-resolution X-ray diffractometry (HRXRD) results proved that the surface step flow movement direction had nothing to do with the off-axis directions of the original substrates. It was found that the surface height difference of substrate was the main driving force for the step flow movement, junction combination and surface morphology changing. The mechanism of the mosaic interface junction combination and step flow transformation on the mosaic surface was proposed.

## 1. Background

The development of diamond growth technology makes diamond a promising material not only in superhard industry, optical components, semiconductor and high power electronics but even also a promising application in the quantum information and computing field [1,2,3,4,5,6]. To realize the actual application of diamond, many researchers focus on the growth of the single-crystal diamond with large size and high quality by the chemical vapor deposition (CVD) method. Due to the low lateral growth rate during the CVD reaction, one can rarely achieve a large single-crystal diamond with small substrates. Although 10 mm × 10 mm single-crystal diamond substrates are commercially available, the size of the CVD single-crystal diamond is still not competitive with other wide gap semiconductor materials, such as SiC and GaN. Furthermore, the quality problem and expensive price of the large diamond substrate also makes it difficult for the further application [7,8].

One of the breakthroughs regarding the size limitation is called “mosaic” technology. It was first proposed by Geis et al. in 1990s and the diamond was grown by a hot-filament CVD system. However, due to the limitation of the system ability, the early mosaic wafers had poor crystal quality with pin holes at the corner and junction boundaries. Yamada et al. created the “Clone” technology to prepare free-standing diamond wafers in the MPCVD equipment and achieved a 2-inch mosaic plate later, but the mosaic boundaries were easily identified by naked eyes and interface crystal quality remained low [9]. Muchnikov et al. studied mosaic CVD diamond crystal growth and paid attention to the crystallographic orientation inherits [10]. No specific correlation between the misorientation of the diamond seed and the substrates junction boundary morphology was found. Shu et al. mapped the stress and defect distributions in the cross-section of the mosaic junctions with Raman spectroscopy, measured and calculated the maximum stress value and corresponding position distance away from the interface [11]. However, few studies focus on the relationship between the surface morphology variation and the internal stress of the junction. Meanwhile, a lack of an evaluation standard for the crystal quality of the junction also increase the difficulty in studying the crystal growth behavior among the mosaic plates. As we know, a uniform step flow generation and movement on the interface junction are quite important to achieve a high quality mosaic connection with low internal stress and little dislocations.

In this paper, we fabricated a CVD single crystal mosaic layer on four seed pieces of high temperature high pressure (HTHP) single crystal substrates. The substrate surface was roughly parallel to (100) plane with a 2–4 °C misorientation. After the CVD growth for 24 and 48 hours, the mosaic top surface morphology was observed by optical microscopy. Internal stress and crystal quality were assessed by Raman spectroscopy and high resolution X-ray diffractometry.

## 2. Experiment

### 2.1. The Preparation of the Diamond Substrates

High-quality Ⅱa type HTHP single crystal diamonds were grown in the cubic press system with a solvent of metal Fe-Ni-Al system in 0.9 GPa by Jinan Diamond Technology Co., Ltd. (Jinan, China) After HTHP growth, the diamond crystals were sawed parallel to the (100) surface with around 3° miscut angle (Please check the text below for the detail angles) by a laser-cutting system [12]. The side faces are polished parallel to (010) or (001) plane which is perpendicular to the growth surface. Mechanical polishing was employed to remove the amorphous and graphite carbon, and the remaining thickness was about 0.3 mm (Table 1). Eventually, the surface roughness average (Ra) reaches to 2 nm confirmed by Atomic Force Microscope (Veeco, Plainview, NY, USA). Before the CVD growth, the substrates were cleaned in a mixture of the sulfuric acid, nitric acid and perchloric acid for three hours. Then they were placed into the acetone and methanol for ultra-sonic cleaning to remove the organic material from the surfaces.

### 2.2. Mosaic Growth of the Chemical Vapor Deposition (CVD) Diamond

The CVD mosaic growth was operated in Arids-300 type MPCVD system (Optosystems Co. Ltd., Moscow, Russia) equipped with a 6 kW/2.45 GHz microwave reactor. In order to increase charged plasma density and the crystal growth rate, closed type substrate pocket holder was used throughout all the reaction. Four pieces of HTHP diamond substrates of 5 mm × 5 mm were placed in the center of the substrate pocket holder next to each other in a 2 × 2 matrix in order to achieve a 10 mm × 10 mm size mosaic plate. Hydrogen etching was operated before the growth to remove the surface impurity and mechanical polishing scratching in 900 °C/300 torr for 30 min. The CVD reaction was performed at 1100 °C/300 torr with a gas mixture of CH_4_/H_2_ at 16/600 sccm flow after the hydrogen etching.

### 2.3. Mosaic Growth Analysis

In the experiment, sample thicknesses were measured before and after 24 and 48 hours’ growth respectively to calculate the growth rate. The top surface morphologies were captured by the confocal laser scanning microscope with the type of Olympus OLS4000 (Tokyo, Japan). Raman mapping for the sample surface was measured by the LabRAM HR800 Horiba Jobin Yvon (Kyoto, Japan) with 532 nm solid laser system at room temperature. The mapping region was located in the center of the sample covering the interface junction and vicinity homoepitaxy area from all the mosaic substrates. Substrate off-axis angle directions and crystal quality were investigated by a high-resolution X-ray diffractometer (HRXRD) by D8 Discover from Bruker (Karlsruhe, Germany). The system was operated at 40 KV and 40 mA with CuK_α1_ radiation. The X-ray was generated as a scattered beam and was aligned parallel by the Goebel mirror to the Ge (220) monochromator. The beam reflected from the mosaic sample was collected by LynxEye detector (Karlsruhe, Germany).

### 2.4. Experiment Result

#### Homoepitaxy Growth and Morphology of the CVD Mosaic Layer

**Figure 1:**
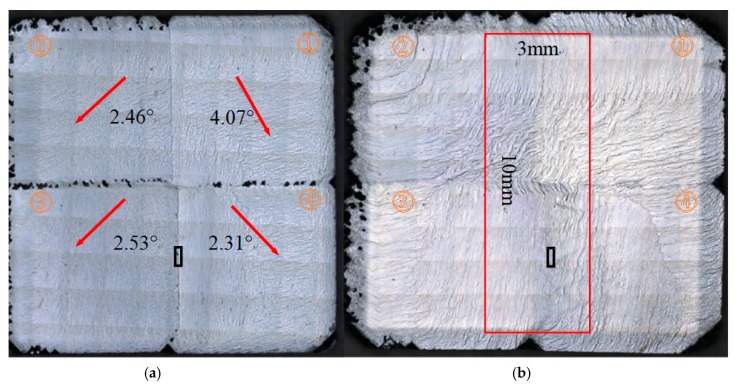
(**a**,**b**) The confocal laser-scanning images of the entire as grown mosaic sample after growth of 24 and 48 hours respectively, the substrate identification numbers are labeled as 1 to 4 in the clockwise direction. The red arrows in Figure 1a corresponded to the off-axis directions of the (100) planes of the original substrate 1 to 4 with the misorientation angles. The red block in Figure 1b shows the region with a diameter of 3 mm × 10 mm area for the Raman mapping measurement, the red lines correspond to the height scanning routine below.

After the CVD mosaic growth, the sample did not show any crack on the junction edge and the entire wafer appeared like a solid plate. The size of the mosaic diamond increased to 10.95 mm × 10.94 mm and 11.75 mm × 11.75 mm after 24 and 48 hours’ growth due to the lateral growth. The Table 1 is the substrates thickness and calculated growth rates of the mosaic after 24 and 48 hours’ growth. The substrate identification number is labeled in the clockwise direction as shown in Figure 1a. The lateral overgrowth rates were calculated to be 16.98 μm/h and 16.25 μm/h while the vertical growth rates of all substrates are about 16–17 μm/h.

Figure 1 shows the confocal laser scanning images of the entire as-grown mosaic sample after 24 and 48 hours’ growth. A clear brighter rim area can be observed near the edge of the mosaic sample in Figure 1b, which refers to the crystal lateral growth region over the original diamond substrates. After the CVD growth, most of the area was covered by regular step flow on the top surface. Small polycrystalline clusters with diameter ranges from 30 μm to 150 μm appeared after 24 hours’ growth on the center junction edges among the substrates, however, they disappeared after growth of 48 hours as shown in Figure 1b. Same phenomena can be observed for the polycrystalline on the lateral growth rim region.

The off-axis direction angles of the (100) faces were also measured by the HRXRD and shown in Figure 1a. The arrows on the four substrates corresponded to the (100) crystallographic axis off directions. Thus, the substrate surfaces were not strictly parallel to the (100) planes instead of having different off-axis angles and directions. However, the entire mosaic surface morphology showed anisotropy step flow domains along the orientations to the substrates off-axis direction after the CVD growth.

**Figure 2:**
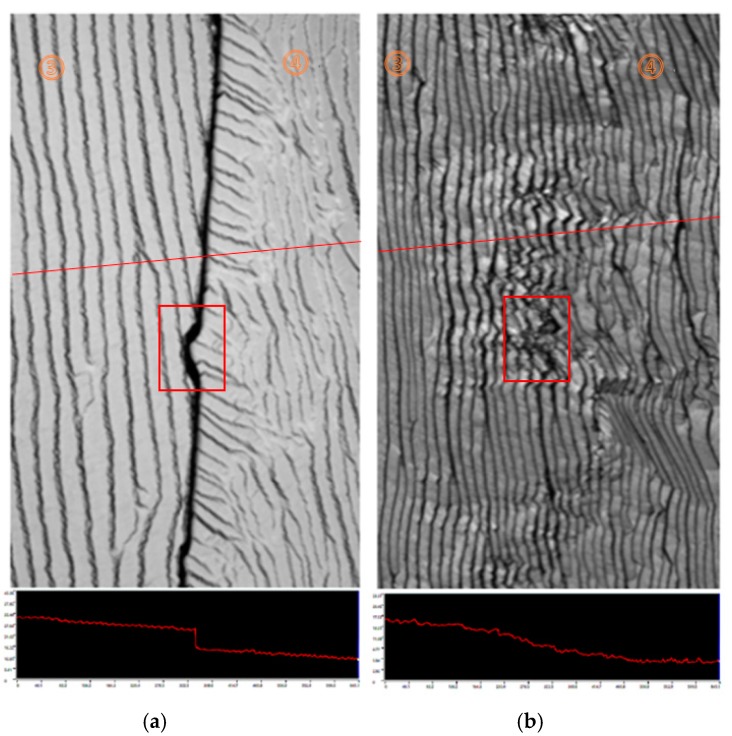
(**a**,**b**) are the images of the junction between the substrates 3 and 4 after the growth of 24 and 48 h from the black block of the Figure 1a,b respectively. The red lines across are the surface height scanning routine and height distributions are attached below.

Figure 2a,b are the images of mosaic interface area between substrate 3 and 4 after the growth of 24 and 48 h with the height scanning images in the lower parts. The junction assumed a “Notch” shape and appeared on the substrate 3 edge. Its length is about 43 μm along the substrate edge. An obvious gap could be found between the two substrates and the height difference was about 9.045 μm. There was a narrow area with the width of 30 μm located in the substrate 4 near the junction area showing a step kink with the rotation angle for about 35° to 50°, which probably corresponded to the lateral growth region of the substrate 1 in the center. After another 24 hours’ growth, the entire junction region was covered by the (100) step with the same step flow direction as shown in Figure 2a,b provides a clear image proving that the gap in the center disappeared. In this figure, the rotation step flow can also be seen clearly. The height scanning image attached also revealed a uniform continuous curve across the junction. The junction “Notch” on the former image transformed to a disturbance of the step flow in the red block. The step flow exhibited a “zigzag” pattern. In contrast, the step flow in other area showed a fluent and uniform pattern.

**Figure 3:**
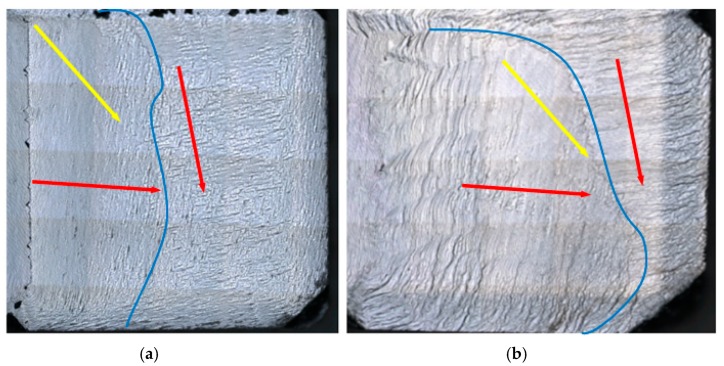
(**a**,**b**) are the surface step flow transformation of substrate 4 after the growth of 24 and 48 hours’ growth. The blue lines mark the boundary of the two surface domains with different step flow directions labeled by red arrows, while the yellow arrows are the (100) off direction of substrate 4.

Figure 3 is the comparison of the surface morphologies from substrate 4 after the growth for 24 and 48 h. One can easily see the transformation of surface step flow directions and domains. The blue lines separate two surface domains marked with red arrows presenting the step flow moving directions. While, the yellow arrows correspond to the off-axis directions of the diamond seeds. Both step flow directions in the two domains are different from the off-axis directions of the original seeds, i.e., the new step flow moving direction has nothing to do with the off-axis direction of the original seed in the procedure of growth. However, the primary step flow almost paralleled to the interface between substrates 3 and 4 “invaded” into the secondary step flow region generated from the original seed from the left to the right. 

**Figure 4:**
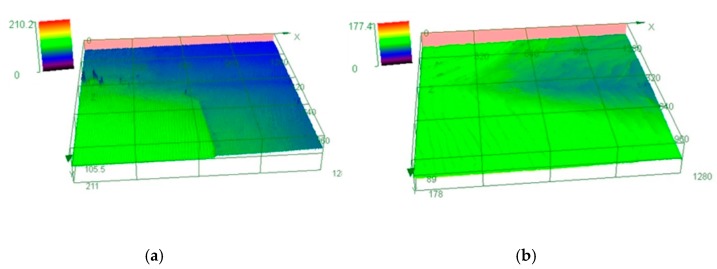
(**a**,**b**) are the height distribution images of the junction center of the substrates after the growth of 24 and 48 hours captured by confocal laser scanning.

Figure 4a,b are the images of the height distribution of the cross junction covering four substrate regions after the 24 and 48 hours’ growth respectively. The images are obtained by the laser height scanning microscope in 1279 μm × 1279 μm area. From Figure 4a, an obvious height gap appeared between the edges of substrate 3 and 4 which matched the result of Figure 2a. However, the height difference of the substrate 1 and 2 junction was difficult to determine. The height order of the four substrates in the interface junction ranks as 3 > 4 >2 > 1. The entire junction area was covered with the step flow along the same direction from the left to the right. The step terrace width ranges from 9 to 12 μm after 24 hours’ growth. However, the junction was covered with the step rotation in the interface of the edge, the step bunching happened while the terrace width broadened to 36–55 μm after growth of 48 hours.

**Figure 5:**
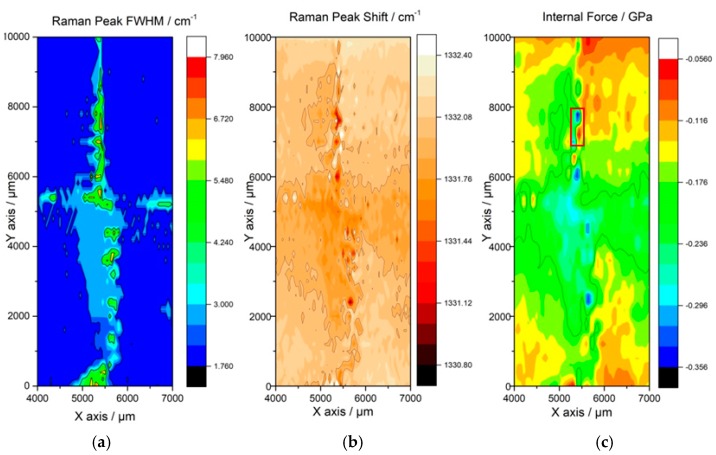
The Raman mapping area of the mosaic sample, the scanning region covered the junction interface edge in the center for the 3 mm × 10 mm region. (**a**) The Raman full width at half maximum (FWHM) of the mapping region, the FWHM ranges from 3.000–8.000 cm^−1^ and verified from the color bright red to deep blue. (**b**) The Raman sp^3^ diamond peak shift of the mapping region, ranging from 1332.37 to 1331.45 cm^−1^. (**c**) The calculation result of the internal stress distribution of the mapping region based on the result of (**b**).

In order to investigate the internal stress and interior defect distribution of the mosaic sample, Raman mapping was performed on the top surface of the center area for the sample after 48 hours’ growth. The mapping region is 10 mm × 3 mm and covers the cross junction of the homoepitaxy layer from the four substrates, as shown in the Figure 1b. The mapping measurement was performed with the scanning step of 50 μm and 200 μm respectively in the X and Y axes, while the integral time lasted 1.2 second for each point. Figure 5a is the full width at half maximum (FWHM) distribution image of the Raman sp^3^ diamond carbon phase whose unstressed Raman peak is about 1332.5 cm^−1^. The result was fitted by Lorentz simulation and exhibited color ranges from 1.760–7.960 cm^−1^. A clear narrow “cross” shape region to a width of about 400 μm was revealed in the center with a larger FWHM than that in the vicinity area, corresponding to the crystal quality distribution on interface junction and the layer grown from the original substrates respectively. The FWHM of Raman peak at the central junction illustrated indirectly that the crystal layer created by the lateral growth in the center was single crystal with acceptable quality. The joint boundary between substrates 3 and 4 moved about 200–400 μm towards substrate 4 because of the step flow movement, which was confirmed by the surface morphology in Figure 2. The FWHM of the Raman peak of the homoepitaxy layer grown from original substrates was smaller and approximate to 3 cm^−1^, which represented the good quality of the crystal layer grown on substrates. The FWHM of the junction area ranges from 3.000 to 8.000 cm^−1^ in the center, only several small areas in the interface showed FWHM larger than 4 cm^−1^. Figure 5b shows the Raman peak shift of diamond sp^3^ peak of the entire mapping area. The peak shift from 1332.37 to 1331.49 cm^−1^, i.e., the peak in all area shifts to the lower wave number in junction area compared with the unstressed diamond peak of 1332.5 cm^−1^. The peak shift mapping distribution also present a “cross” pattern similar with the FWHM distribution which shows a higher wave number shift from the center to the periphery area. Steep discoloration can be observed in several areas on the central junction and the peak shift changed for about 1–2 cm^−1^. Same spots appeared on the largest peak shift area between the substrate 3 and 4 moves to the direction of substrate 4 for about 200–400 μm which match the FWHM distribution image in the Figure 5a. 

Figure 5c shows the internal stress distribution of the scanning area. The internal stress was calculated by the following formula according to the result of Figure 5b:

$$P=0.34\frac{GPa}{cm^{-1}}\times \left(\upsilon -\upsilon_{0}\right)$$

where $\upsilon_{0}$ refers to the theoretical value of the unstressed diamond layer Raman peak, normally is 1332.5 cm^−1^. υ is the measured position of the Raman peak [13].

According to the calculated result of Figure 5c, the internal stress of the mapping region ranged from −0.356 to −0.056 GPa. Therefore, the whole region was filled with a tensile stress. The central area between substrate 1 and 2 shows the strong stress fluctuation, meaning the release of the accumulated tensile stress. The largest difference value of the stress is about 0.295 GPa from the red block in Figure 5c.

**Figure 6:**
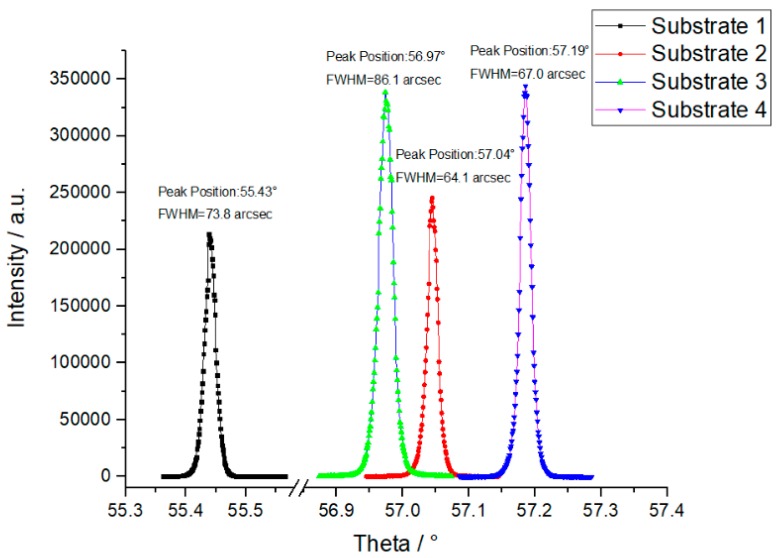
The high-resolution X-ray diffractometry (HRXRD) rocking curves of the (400) reflections from four positions on the mosaic homoepitaxy layer grown on the different substrates, the peak positions reflected the misorientation of the original substrates.

In order to understand the evolution of the surface morphology and assess the layer crystal quality, HRXRD was used to measure the rocking curves of (100) reflection in the different positions of the mosaic sample. The FWHM of rocking curve reflects homoepitaxy layer quality. Figure 6 shows the rocking curves of the (400) reflection measured from the mosaic layer grown from four substrates. All the measuring results show a single narrow rocking curve peak and the position of the angle ω varies from 55.43° to 57.19° which corresponded to a 2.31° to 4.07° misorientation generated from the cutting and polishing. The FWHM are in the range of 60–90 arcsec, which reveals that the homoepitaxy layer has good quality. However, substrate 2 and 3 possessed the smaller misorientation angles than substrate 1 and 4, the step flows on substrate 2 and 3 showed the most aggression for the movement of the interface in the center area after the CVD growth.

## 3. Result Discussion

The above diamond homoepitaxy layers are different from those grown by the “clone” method. The key of the “clone” technology is to apply the exact one single diamond plate to create numbers of repetitive substrates with same crystallographic morphology on the top surface for the mosaic growth to avoid crack of the junction boundary. However, unlike the method for “clone” technology, the off-axis directions of substrates in our experiment were not controlled to the same crystallographic direction before the growth since they may be not from one HTHP diamond. By contrast with the report from Hideaki Yamada et al., we did not see the similar morphology and crack on the mosaic junction area, since the angle between two off-axis directions for the substrate 3 and 4 was about to be 90°. The off-axis direction from the original substrate did not affect the surface step flow direction for the CVD mosaic growth [14,15]. The observation on the surface morphology in our experiment indicated that the step flow across the central junction showed more correlation with the thickness of all substrates used in mosaic growth.

As we know, unlike other crystal growth, such as liquid phase or melting method, MPCVD for diamond growth is a method with growth rate violently influenced by the plasma generated by the microwave and gas mixture instead of temperature gradient and material concentration. The single diamond CVD deposition is a continuous reaction in a stable hydrogen/methane environment with a self-coupled electromagnetic field. The chamber shape and gas pressure play an important role in the transmission of the microwave to create plasma with acceptable shape and density for the carbon deposition. The surface morphology could present a uniform step flow along the same direction covering the entire substrate with an excellent control in the system parameters [16,17,18].

Many studies have focused on the environment design for the plasma generation and deposition condition, including the shape of the chamber, gas pressure and substrate pocket holder [1,19,20,21,22]. Several reports simulated the substrate surface condition and found that the plasma-discharged electric field intensity and surface temperature were much higher on the surface for the thicker substrates than the thinner one [20,23,24]. This implies that more carbon ions with higher free energy may overcome the potential barrier to deposit on the diamond substrate when a thicker substrate is used. It causes an excessive high growth rate compared to the lower area and generates the new step flow with the direction perpendicular to the contour line of height distribution. 

However, for the MPCVD diamond mosaic growth, more parameters should be considered, such as the gap width, substrate shape and thickness difference. In this way, the substrate surface morphology could receive secondary effects introduced by the other substrates during the mosaic growth. A new step flow may generate along the different direction over the original one when the substrate edges meet each other or polycrystalline was grown in the junctions [25,26]. Shu et al. studied the stress and defects distributions in the cross-section of the mosaic junctions with Raman spectroscopy, measured and calculated the maximum stress value and corresponding position distance away from the interface [11,27]. They reported that the location of the major part of the defected zone might move from one crystal to the neighbor crystal across the junction, which matches our result in Raman surface mapping and the surface step flow movement. 

In previous research, it was found that the internal stress of the diamond homoepitaxy growth layer could be inherited from the defects of substrate [28,29]. The substrate defects could be introduced from the internal inclusions of the HTHP single-crystal diamond or the cutting and polishing process after the growth. Anatoly B. Muchnikov et al. pointed out that the surface stresses generated by the thermochemical polishing should be also taken into consideration for the formation of the surface step direction when preparing the diamond mosaic [10]. We believe that the scratches might introduce an anomalous charge ion concentration and temperature gradient on the substrate surface. As a result, step flow direction may be changed. In our experiment, a high-quality HTHP diamond was used for the substrates preparation. The fine polishing was performed after the laser cutting and a scratching-free upper and side surface with Ra smaller than 2 nm was achieved. In this way, the internal stress in both the homoepitaxy and lateral growth area would be reduced during the CVD growth since anomalous charged electric field on the deposition surface and edges was avoided in a smooth surface. As a result, the central area eventually shows the uniform step flow without any cracking even though the clear height gap appeared after the mosaic growth for 24 hours.

### Mechanism of the Mosaic Junction Creation and Interface Step Flow Movement

**Figure 7:**
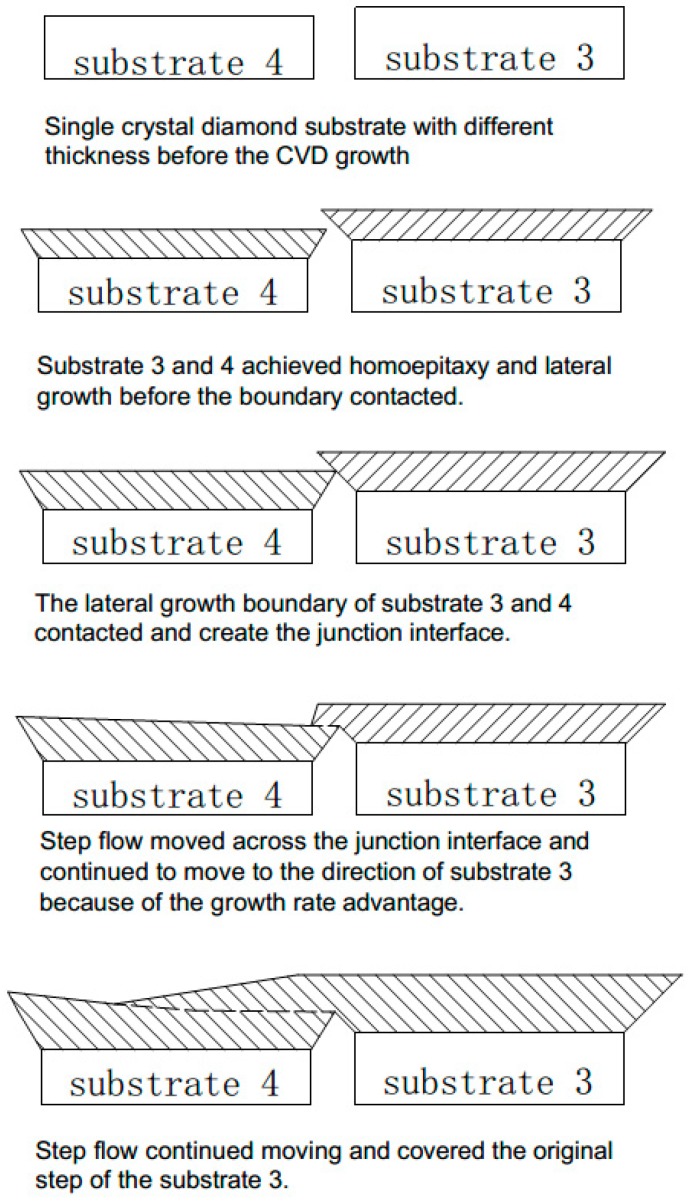
The mechanism of the CVD diamond mosaic junction interface formation and the movement of the surface step flow.

Based on the surface morphology, Raman scanning and HRXRD result of the substrate interface, we schematically proposed the mechanism to describe the combination of high quality mosaic interface junction and the step flow movement and transformation across the junction on the deposition layer as shown in Figure 7. Before the growth, the substrates were placed into the reaction chamber, with narrow gap space among the diamond seeds. Even with carefully cutting and polishing, thickness differences still existed among them. When the CVD reaction started, a homoepitaxy growth occurred on the substrate surface and the original step flow was formed, which is normally caused by thickness difference of the substrate surfaces. Simultaneously, lateral overgrowth happened on the substrates edge and the gap distance between the substrates reduced persistently when the deposition proceeded. With continuous lateral growth, the two edges contact and form interface junctions between the substrates. Meanwhile the homoepitaxy growth causes the increase of the sample thickness, the mosaic shall penetrate into the plasma in the chamber. As a consequence, the charged electric field density will increase on the edges and corners of the sample, which results in a higher temperature and the concentration of charged carbon on the interface. This leads to the slightly increase of the crystal growth rate in center junction and the formation of the new step flow parallel to the interface and perpendicular to the original surface step. The new step flow moves across the gap and consolidates the two individual substrates into an entire block plate. After the combination of the interface junction, the new step continues to move on the samples and cover the original step flow, and the whole mosaic plate surface was eventually covered by a uniform surface step.

## 4. Conclusions

In this paper, a mosaic diamond was grown by MPCVD on HTHP single-crystal diamond plates. After diamond growth, we observed the morphologies of the mosaic edges and center junction and analyzed the step flow movement across the junction interface. Raman mapping images proved that the mosaic layer in the center junction possessed high crystal quality and the junction stress field moved for about 200–400 μm from one substrate to another. The HRXRD result indicated that the mosaic surface step flow direction had nothing to do with the off-axis direction of the original substrates. Furthermore, the surface height distribution proved that the surface step flow generation and movement was driven by the height difference of the entire mosaic surface. The mechanism of the junction interface formation and the mosaic surface step morphology transformation was proposed.

## Figures and Tables

**Table 1:** Thickness and calculated growth rate of the mosaic sample before and after chemical vapor deposition (CVD) growth.

**Thickness (mm)**	**Substrate 1**	**Substrate 2**	**Substrate 3**	**Substrate 4**
Before Growth	0.27	0.28	0.32	0.27
Growth 24 h	0.65	0.7	0.72	0.69
Growth 48 h	1.07	1.09	1.13	1.09
**Growth Rate (μm/h)**	**Substrate 1**	**Substrate 2**	**Substrate 3**	**Substrate 4**
Growth 24 h	16.18	17.71	17.61	16.77
Growth 48 h	17.50	16.25	17.08	16.67
